# Enrichment of homologs in insignificant BLAST hits by co-complex network alignment

**DOI:** 10.1186/1471-2105-11-86

**Published:** 2010-02-12

**Authors:** Like Fokkens, Sandra MC Botelho, Jos Boekhorst, Berend Snel

**Affiliations:** 1 Theoretical Biology and Bioinformatics group, Department of Biology, Faculty of Science, Utrecht University, Padualaan 8, Utrecht, 3584CH, Utrecht, the Netherlands; 2 PhD Program in Computational Biology, Instituto Gulbenkian de Ciência, Oeiras, Portugal; 3 Netherlands Bioinformatics Centre, Geert Grooteplein 28, Nijmegen, 6525 GA, the Netherlands

## Abstract

**Background:**

Homology is a crucial concept in comparative genomics. The algorithm probably most widely used for homology detection in comparative genomics, is BLAST. Usually a stringent score cutoff is applied to distinguish putative homologs from possible false positive hits. As a consequence, some BLAST hits are discarded that are in fact homologous.

**Results:**

Analogous to the use of the genomics context in genome alignments, we test whether conserved functional context can be used to select candidate homologs from insignificant BLAST hits. We make a co-complex network alignment between complex subunits in yeast and human and find that proteins with an insignificant BLAST hit that are part of homologous complexes, are likely to be homologous themselves. Further analysis of the distant homologs we recovered using the co-complex network alignment, shows that a large majority of these distant homologs are in fact ancient paralogs.

**Conclusions:**

Our results show that, even though evolution takes place at the sequence and genome level, co-complex networks can be used as circumstantial evidence to improve confidence in the homology of distantly related sequences.

## Background

Comparative genomics involves large scale investigations to identify which parts of different genomes are of common descent in order to predict function or to study genome evolution. A common first step towards detecting homology between genes or proteins within a genome or between different genomes, is to do a BLAST search with a set of genes or proteins against a database and regard each hit with an E-value below a certain cutoff to be homologous [1]. Additionally, several filters and clustering algorithms can be applied to separate sets of homologs into orthologous groups (e.g[2,3]). Usually, a stringent score cutoff is used to ensure that the hits that are included are indeed homologs. Naturally, homologs whose sequences have diverged strongly, are incorrectly excluded.

On a smaller scale, more sensitive searches based on profiles of groups of related amino acid sequences (such as PSI-BLAST or HMMer) or, if available, protein three dimensional structures are commonly used to avoid False Negatives without losing confidence in the putative homologs returned [4,5]. In these searches, instead of using the same scores or probability for each position, a multiple sequence alignment is used to define position specific substitution scores or transition probabilities.

Besides improving sequence based homology searches, one can also use information on the genomic context of sequences to aid detection of a common descent of sequences. Genome alignments can be very useful when there are difficulties in determining homology between sequences, for example between intergenic regions. Boekhorst and Snel showed that genome alignments can be used to select candidates from a set of insignificant BLAST hits in prokaryotes [6]. In eukaryotes, gene order is less conserved across large phylogenomic distances such as between fungi and animals and therefore less likely to make a valuable contribution to the detection of homology at these large evolutionary distances [7]. As a result, conserved synteny is mainly employed in eukaryotes for the detection of orthologs, between closely related species, e.g. within ascomycete fungi or within vertebrates [8,9].

**Figure 1:**
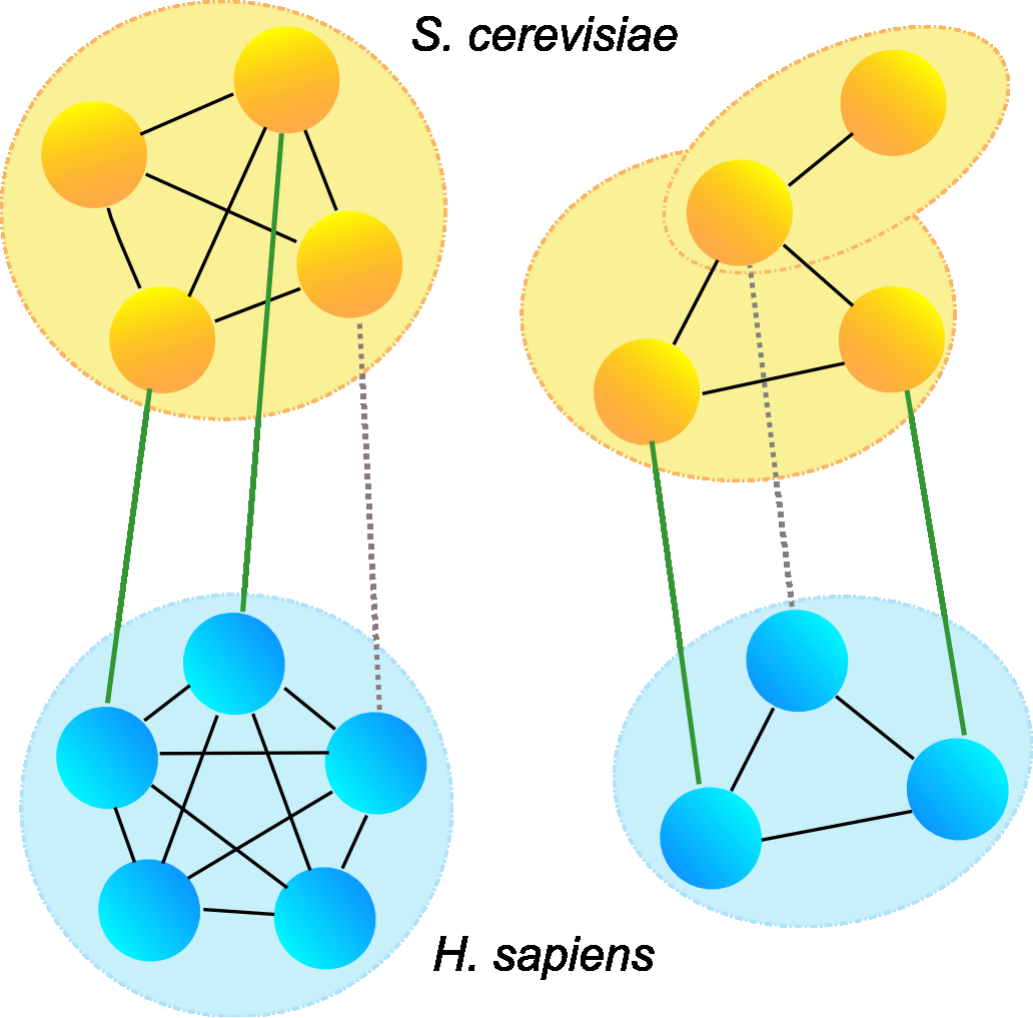
**Co-complex network alignment and homology inference in insignificant BLAST hits**. Green lines: human-yeast unambiguous and readily identifiable orthologs (human and yeast proteins in one Inparanoid cluster), gray dotted line: insignificant BLAST hit. If two proteins with an insignificant BLAST hit are subunits of homologous complexes, are these proteins more likely to be homologous than would follow from the score returned by BLAST?

The availability of protein interaction networks allows for the comparison of genomes and the functional context simultaneously. Information on the functional context of proteins is already used in comparative genomics of eukaryotes to select from a set of inparalogs, the protein that is functionally similar to the query sequence (the 'functional ortholog') [10-12]. In the comparative analysis of protein interaction networks, spurious protein interactions can be separated from biologically relevant interactions if the protein-protein interaction occurs in different species. We here test if the reverse is also in principle applicable: can the network alignment help to separate spurious homology links from real ones? Analogously to genome alignment, we test the expectation that the insignificant blast hit between protein *a *from species A and protein *b *from species B is more likely to reflect homology if protein *a *is functionally closely related to proteins which are readily identifiable as orthologous to proteins in species B that are functionally closely related to *b *(Figure 1). To answer this question, we select candidate pairs from BLAST hits between human and yeast proteins based on conserved functional context, in this case homologous complexes, and determine whether this selection contains relatively more homologs than a background of hits with similar BLAST scores.

## Results and discussion

### Are hits with conserved functional context more likely to be homologous?

We perform an all-against-all BLAST search between the human and yeast proteomes using a substantially more inclusive threshold than normally is applied to allow a comprehensive survey of insignificant BLAST hits. For each query-hit pair BLAST returns an E-value. We bin the E-values into 8 bins ranging from [E < = 10^-5^] to [10 < E < = 100]. We define co-complex networks for human and yeast based on two curated complex datasets per species, and use Inparanoid clusters between human and yeast to align these networks (Figure 1 and Methods) [2,13-15]. If the query and the hit contain a domain which belongs to the same Pfam clan, we consider them to be True Positives. For each of our 8 bins, we calculate the fraction of query-hit pairs which are True Positives, with and without co-complex network alignment.

**Figure 2:**
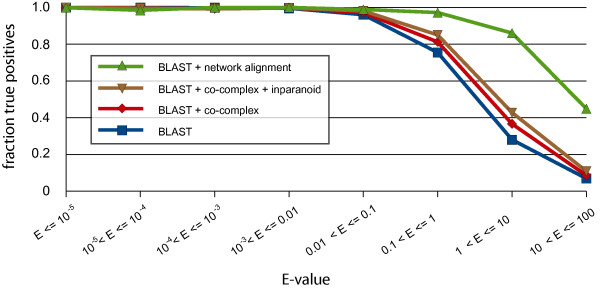
**Fraction of True Positives for different E-value bins for different subsets of BLAST hits with that E-value**. The fraction of True Positives for all BLAST hits ('BLAST', blue line), the BLAST hits for which both the human query as the yeast hit are part of a co-complex network ('BLAST+cocomplex', red line), the BLAST hits for which both the human query as the yeast hit are part of a co-complex network and both have a direct co-complex network neighbour that has a clear ortholog in the other species (is part of a human-yeast Inparanoid cluster) ('BLAST+cocomplex+inparanoid', brown line), the BLAST hits for which both the human query as the yeast hit are part of a co-complex network and both have a direct co-complex network neighbour and these neighbours are clear orthologs of each other (are part of *the same *human-yeast Inparanoid cluster) ('BLAST+network alignment', green line).

We find that the use of co-complex information results in a considerable increase in the fraction of true homologs among the returned hits, compared to BLAST without co-complex information (Figure 2). This difference is most eminent in bins representing E-values normally considered to be insignificant (the 'gray-zone'). At E-values between 1 and 10 almost 90% of the returned hits share a Pfam clan, which means a substantial, 8 fold increase in the percentage of True Positives. This is not due to a bias resulting from being a member of the co-complex network or being in a conserved region of the co-complex network, as only after *alignment *of the co-complex network we see a big improvement in the fraction of True Positives (Figure 2).

Only a small subset of yeast and human proteins (~12% of human and ~26% of yeast proteins) is part of a co-complex network within each species. Moreover, many of those are not functionally linked to proteins that have readily identifiable orthologs in the other species. As a consequence, this method is applicable to only a small fraction of query-hit pairs (Table 1). If we include high-throughput co-complex datasets for yeast and human, the coverage is increased a little at a cost of a slightly inferior performance (see Additional file 1).

**Table 1:** Applicability

BIN	BLAST		Co-complex network alignment	
	**Number of pairs**	**Number of queries**	**Number of pairs**	**Number of queries**

E < = 10^-5^	180299	16271	1599	791

10^-5^ < E < = 10^-4^	19238	6771	102	88

10^-4^ < E < = 10^-3^	15916	6566	102	86

10^-3^ < E < = 0.01	23882	8626	152	118

0.01 < E < = 0.1	27273	10818	164	122

0.1 < E < = 1	53861	22861	192	155

1< E < = 10	233649	44138	288	222

10 < E < = 100	1108105	46427	787	495

Each row contains the number of query-hit pairs and the number of distinct human query proteins in a particular E-value bin, for all BLAST hits and the subset of BLAST hits which falls into the 'co-complex network alignment' category.

We show that alignment of co-complex networks can facilitate the identification of true homologs among gray zone BLAST hits. In a simple and completely automated procedure, we obtain a subset of hits which, despite very high E-values, is substantially enriched for homologs. This allows us to infer homology for pairs with co-complex network alignment with an E-value ranging between 0.1 and 1 with the similar confidence as for pairs before co-complex network alignment and an E-value of 0.01 (Figure 2). Our framework would likely be improved if we could use statistics on (locally) missing connections in both co-complex networks. To date, protein complex datasets are too fragmentary to make any sensible estimates of the number of missing connections.

### Detection of missing complex subunits

Previous large scale investigations towards presence and absence of protein complex subunits in prokaryotes and eukaryotes reveal that most complexes are only partially present in other species [16-18]. In these studies, an orthology definition based on BLAST is used to determine presence and absence of subunits in different species and part of the subunits regarded absent may be missing due to detection problems. Hence the disrupted co-evolution of protein complexes might partly be an artefact.

The use of co-complex information is potentially useful in the detection of yeast homologs of subunits of human protein complexes. Especially as the most important disadvantage, the lack of coverage of the co-complex networks, is less urgent because the queries are subunits and hence are all part of the human co-complex network. We take the opportunity to test the applicability of our method to a problem in comparative genomics and assess the added value of co-complex network alignment in detecting homologs in yeast for subunits of human complexes. For the complexes in the CORUM dataset, we initially find homologs for complex components by running a BLAST search with all subunits against the yeast proteome, applying a commonly used E-value cutoff of 0.001. Then, for the subunits we did not find homologs for, we use a less stringent E-value cutoff of 1, in combination with co-complex network alignment, to see how many additional subunits we pick up.

Using BLAST only with an E-value cutoff of 0.001, we find yeast homologs for 1199 out of 1901 (63.07%) subunits. We find that 172 out of 710 complexes (24.23%) have a homolog in yeast for all subunits, 427 (60.14%) have homologs for some subunits and 111 (15.63%) complexes are completely absent. Even when only comparing two species, we find that for most human complexes, only part of all subunits have a homolog in yeast. However, as we have argued before, some subunits may be called absent due to detection problems.

For the 702 subunits for which we detected no homolog in yeast, we select, using the co-complex network alignment, candidate homologs in yeast for 52 additional subunits, belonging to 62 complexes (some subunits are part of multiple complexes). Using Pfam, CDD and PSI-BLAST, we confirm that the 49 out of 52 candidates recovered with co-complex network alignment are in fact homologous. With the 49 confirmed homologs we retrieved, an additional 19 complexes are completely present in yeast (see Additional file 2).

One striking observation when comparing individual complexes in human to complexes in yeast, is that there is very little congruence between human and yeast complex definitions (see Additional file 3). Factors such as the incompleteness of data in both species, individual decisions on what does belong to a complex and what does not, and proteins belonging to multiple complexes, obscure a one-to-one relation between yeast and human complexes, assuming such a correspondence exists.

**Figure 3:**
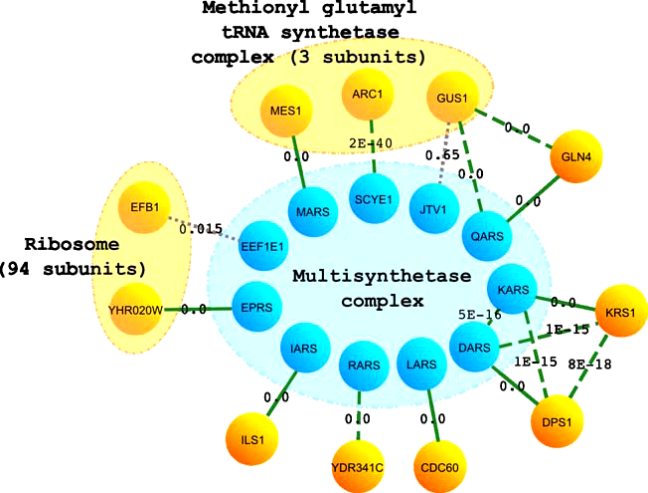
**The Multisynthetase complex**. Yeast homologs were detected for all subunits of the Multisynthetase complex. Green solid lines link proteins which are together in an Inparanoid cluster, green dashed lines indicate a significant BLAST hit between the two proteins linked, gray dashed lines indicate insignificant BLAST hits between proteins for which homology is confirmed by the co-complex network alignment.

Fortunately, because we align human and yeast complexes on a network level rather than as individual complexes, we are able to retrieve homologs with the co-complex network alignment for complexes which do not exist as such in yeast. A good example is the Multisynthetase complex (Figure 3). This complex is composed of 8 aminoacyl-tRNA synthetases and 3 auxiliary proteins. The individual tRNA synthetases all have a homolog in yeast found with the initial straightforward BLAST search. The yeast homologs of the tRNA sythetases are not known to be organized in a complex, with one important exception: methionyl and glutamyl synthetases MES1 and GUS1 associate into a complex with ARC1, an auxiliary protein (and homolog of the human auxiliary protein p43, SCYE1) which increases catalytic efficiency and ensures correct localization into the cytoplasm. Via this complex, human JTV1, a scaffold required for the assembly and stability of the multi-tRNA synthetase complex, is linked to a short N-terminal stretch of yeast GUS1, whose C-term is unambiguously homologous to the glutaminyl synthetase in human, QARS (3). When we do a PSI-BLAST with human JTV1 as a query protein, we retrieve GUS1, aligned to the GST_C domain in JTV1 (E-value 1e-05) after three iterations.

We recovered a homolog for another subunit of the Multisynthetase complex via an unrelated complex: the Ribosome. Human EEF1E1 has a hit with yeast EFB1 with an E-value of 0.015. EFB1 is located at the ribosome, as is YHR020W, which is the readily identifiable yeast ortholog of the human bifunctional glutamyl-prolyl tRNA synthetase EPRS. Both EFB1 and EEF1E1 are translation elongation factors (EEF1E is a translation elongation factor 1 epsilon and EFB1 a translation elongation factor 1 beta) and both contain a domain which belongs to the GST_C_superfamily. The HSP lies in the regions where the GST_C_superfamily domain lies in both proteins and these regions in the protein sequences are, albeit very distantly, evolutionary related. EFB1 has a much more similar homolog in human (namely EEF1B2, BLAST E-value 1e-33), suggesting that EEF1E1 and EFB are related through a very old duplication event and the translation elongation factor 1 epsilon EEF1E1 ortholog is lost in yeast.

Applying the co-complex network alignment to the set of protein complex subunits in CORUM, we select candidate homologs in yeast for 52 proteins, out of which we could confirm homology with Pfam, CDD or PSI-BLAST for 49 pairs. The observation reported in both large and small scale investigations [16-20], that most complexes are 'incomplete' in many species, remains unchallenged because we can only show for a few complexes that their incompleteness is a result of an undetected homology

### Are the recovered distant homologs orthologs?

Exploiting the co-complex network alignment we find yeast homologs for 52 subunits of human complexes that are not revealed by standard BLAST. This is markedly less than the 405 human queries for which co-complex information is applicable (Table 1). The likely crucial difference between our initial survey of all BLAST hit pairs and the detection of missing complex subunits is the fact that in the latter, we applied the co-complex alignment only to those query proteins for which we could not find a homolog with BLAST alone. Therefore we expect that many query proteins for which we recover a distant homolog with the co-complex network alignment in the initial survey, have an additional, significant hit in yeast and are therefore not used as a query when looking for additional homologs for complex subunits. Indeed, we find that this is the case for no less than 85% (347 out of 405) of the query-hit pairs with an E-value > 0.01.

**Figure 4:**
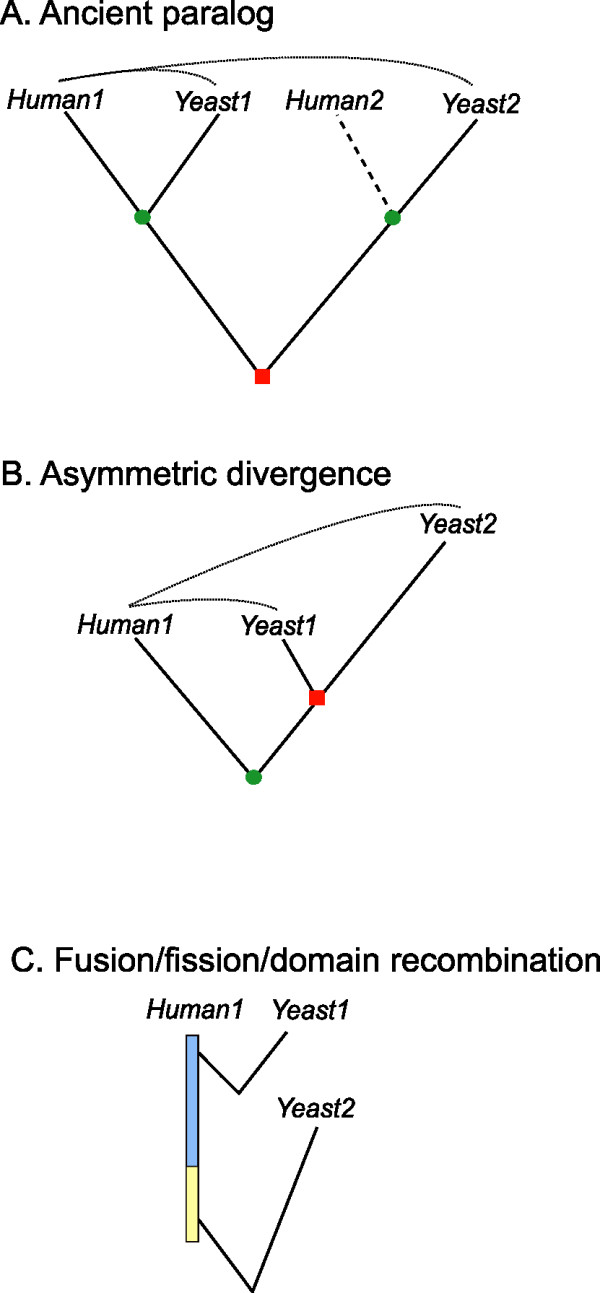
**Evolutionary histories that explain why for a query protein in human, we find both a close and a distant homolog in yeast**. Some proteins for which we recover a distant homolog in yeast with our method, in fact have a better hit (a closer homolog) in yeast. The three scenario's depicted here could have this effect. We test which scenario occurs more often by looking whether the distant homolog in yeast (Yeast2 in this Figure) have a closer homolog in human than Human1. Red square: gene duplication event, green circle: speciation event.

There are a few possible evolutionary histories that can explain the fact that for a certain query protein in human, we find a close homolog and a distant homolog with conserved functional context in yeast. First of all, distant homologs recovered by co-complex network alignment could be ancient paralogs (outparalogs with respect to branching of fungi and metazoa), in which the high degree of divergence is due to time rather than rapid sequence evolution (Figure 4a). For instance, EEF1E1 and EFB1 in the Multisynthetase example discussed above are ancient paralogs. Another possibility is a more recent duplication in yeast followed by asymmetric divergence in the duplicates, in which case the divergence is caused by accelerated evolution on one branch (Figure 4b)[12,21]. Finally, the two yeast hits may be homologous to different regions of the query protein due to fusion, fission or domain recombination events (Figure 4c), in which one domain/region has a markedly higher rate of sequence evolution than the other.

In the fusion/fission/domain recombination scenario, the two yeast hits of the human query protein in yeast are not homologous. For 29 of our 347 trios the two yeast hits are not a significant hit in BLAST, neither do they share a homologous domain according to Pfam. For 27 of these trios the best scoring BLAST HSP of the two yeast proteins is in a different region in the human protein. In the remaining two pairs, the distant homologs that we retrieve share only a short KOW motif with the query protein, while the best hit shares both the KOW motif (not recognized by Pfam, but part of the HSP) and also the adjacent Ribosomal L27e domain.

If we consider only those 318 trios of proteins in which the two yeast proteins are homologous according to Pfam, we find that in 307 of them, the distant homolog has a significant hit in human, suggesting it is in fact an ancient paralog (Figure 4a). A recent study towards the fate of duplicated protein complex subunits showed that 31% of duplicates resides in different complexes, 31% stayed in the same complex and in 38% of the cases one of the duplicates is not known to be part of any complex [22]. We investigate the fate of the 307 yeast pairs that are outparalogs according to our analysis. The yeast pairs are not a random sample of ancient yeast duplicates, on the contrary. Because one of the yeast paralogs is a close homolog of a human protein which is part of a complex which is homologous to the complex the other yeast paralog is part of, we expect a bias towards duplicates remaining in the same complex.

We find that for 139 pairs (45.3%), both duplicates are in the same complex, for 25 pairs (8.14%) the duplicates are in overlapping complexes (sharing more than half of their subunits), for 108 pairs (35.2%) they are in a different complexes and for 35 pairs one of the duplicates (the one most closely related to the human query protein) is not known to be part of any complex in yeast. We expect that the human homologs of the 108 pairs in which the yeast ancient duplicates belong to distinct complexes, are more often part of multiple complexes, indicating that the yeast duplicates subfunctionalized. We do observe a significant overrepresentation of proteins that are part of multiple complexes in ancient paralogs when compared to all subunits in the CORUM complex dataset (P = 0.007), but not in ancient paralogs which are part of the same complex when compared to those which have ended up in distinct complexes (P = 0.58).

The lion's share of distant homologs we recover using the co-complex network alignment consists of ancient paralogs rather than orthologs. Duplications in general are very important in the evolution of protein complexes [22,23] and many structures are known to consist of subunits resulting from very old duplications (e.g. the proteasome). We find that in most cases both duplicates are part of the same or overlapping complexes. This suggests that the duplicates we detect have sub- or neofunctionalized within one complex, although some might be the result of outparalogs that have been independently recruited to a biological process.

## Conclusions

We test whether contextual information from the functional network, in this case conserved co-complex relations, can aid homology detection. Functional context information has been used before to help in choosing functional orthologs from a set of inparalogs, but to our best knowledge, this is the first time functional networks are used to aid distant homology detection. Using an aligned co-complex network, we can identify a subset highly enriched for homologs of BLAST hits with an E-value which would normally be regarded as insignificant. This shows that, even though evolution takes place at the sequence level, one can use co-complex networks as circumstantial evidence to improve confidence in the homology of distantly related sequences.

The interspecies co-complex network includes only a small fraction of all proteins, which impedes applicability. As more high-throughput datasets become available in more species, we expect that the proof of principle we established here, can be applied and tested on a larger scale, between more distantly related species and with other types of functional relations. We apply our co-complex network alignment to a dataset of human complexes in order to determine how many homologous subunits we can detect that we missed in an initial BLAST search. We thereby recovered homologs for only a few additional subunits, despite the fact that coverage is less a limiting factor in this context. We find that one reason we retrieve less additional subunits than expected, is that with the co-complex alignment, we mainly detect outparalogs rather than orthologs.

It has been shown that subunits of a protein complex diverge at similar rates, presumably because subunits of a protein complex are functionally strongly interdependent and subject to very similar evolutionary constraints [24]. In contrast, the co-complex network alignment method is based on the fact that some subunits diverged between human and yeast to such an extent that they are not picked up in a regular BLAST search and other subunits are conserved such that the human and yeast orthologs are still detected by Inparanoid. In this light it is not surprising that most homologs we recover with our method are ancient paralogs rather than orthologs: the difference in the extent of divergence is due to difference in time, as opposed to difference in evolutionary rates between subunits of the same protein complex.

Researchers studying the evolution of individual protein complexes have used functional information to find diverged homologs successfully despite absence of proof from a large scale study [25,26]. Our results provide this proof. Numerous predictions made in these small scale studies were subsequently confirmed by profile vs profile alignments or the comparison of protein three dimensional structures upon availability. Interestingly, many of these predictions represent initial BLAST hits with E-values even higher than 100 (the cutoff used in this study). Hence it is possible that in our study still many homologs have gone undetected, and the formal integration of functional context with more sensitive homology detection methods might help in the development of automatic bioinformatic methods to uncover these distant homologs and improve our insights into the ancient evolution of the protein interaction network.

## Methods

### Co-complex network

To construct a human co-complex network, we download the set of CORUM Core complexes from http://mips.gsf.de/genre/proj/corum and stored the complexes as sets of co-complex pairs [15]. We added 'direct complex' pairs downloaded from Reactome http://www.reactome.org/download/current/homo_sapiens.interactions.txt.gz[13], which, in combination with pairs from the CORUM dataset, results in a co-complex network containing 32415 unique pairs in total. For the yeast co-complex network, we stored MIPS complexes from ftp://ftpmips.gsf.de/yeast/catalogues/complexcat as binary co-complex relationships [14] and complexes from SGD GO cellular component annotation [27] as in [22]. This resulted in 20075 unique pairs in total.

### BLAST and Pfam

We downloaded 46704 human protein sequences from Ensembl [28], (Homo_sapiens.NCBI36.50.pep.all.fa) and yeast protein sequences from the Saccheromyces Genome Database (orf_trans_all.fasta) in July 2008. We run BLAST between human and yeast with the maximum returned E-value set to 100, maximum number of hits and alignments set such there it is no limiting factor [1]. We did not adjust the database size. If two proteins have multiple HSPs (regions aligned by BLAST), we keep only the HSP (*High Scoring Sequence Pair) *with the lowest E-value. We downloaded Pfam HMMs (version 23, July 2008) and data on homologous Pfam families (Pfam clans) from the Pfam website ftp://ftp.sanger.ac.uk/pub/databases/Pfam, searched for domains in human and yeast proteins using hmmpfam in the HMMer package [29] with default cutoffs.

For each BLAST hit, for both the human query protein and the yeast hit, we determine the overlap between the HSP and each Pfam domain and divide the number of amino acids in the overlap with the length of the shortest region (either Pfam domain or HSP) to get a percentage of overlap. If a query and a hit have a Pfam domain that belongs to the same clan and the overlap of the domain and the HSP is greater than 50%, we call this BLAST hit a True Positive. BLAST hits for which this overlap is less than or equal to 50% in either the human query or the yeast target protein are ignored as the gold standard for homology (Pfam clans) can't be fully applied to these proteins.

### Co-complex network alignment

To align the co-complex networks of yeast and human we look for yeast orthologs for all proteins in the human co-complex network using Inparanoid. We run Inparanoid 3.0 with default parameters, so for each bidirectional best hit which forms a seed pair for an Inparanoid cluster, it is required that the minimum BLAST bitscore is 50 and the overlap of the alignment relative to the shortest of the two proteins is at least 50% [2].

For each BLAST query-hit pair, if the human query protein has at least one direct neighbour in the human co-complex network that is orthologous (in one Inparanoid cluster) to the direct neighbour of the yeast hot protein in the yeast co-complex network (Figure 1), we assign this pair to the 'co-complex network alignment' category (Figure 2). We bin E-values in 8 bins ranging from [E < = 10-5] to [10 < E < = 100] and calculate for each bin the percentage of True Positives (hits that each have a Pfam domain belonging to the same clan), also known as the Positive Predictive Value. Each bin contains at least 60 pairs and at least 50 query proteins (Table 1). Normalizing for family size gives similar results (see Additional file 4).

### Detection of missing complex subunits

To avoid biases due to overlapping complexes as much as possible, we removed 803 complexes which are a subcomplex of another complex from the set of CORUM Core complexes. If we remove all supercomplexes instead of all subcomplexes, we get qualitatively the same results. We first attempt to find a yeast homolog with BLAST and an E-value cutoff of 0.001 for all subunits. Subsequently, on those subunits we did not find a homolog for, we applied the co-complex network alignment with an adjusted E-value of 1 (expected percentage of False Positives < 3% (Figure 2)).

## Authors' contributions

BS conceived the study and assisted in writing the manuscript. LF, SB and JB performed the analysis and LF wrote the manuscript. All authors have read and approved the manuscript.

## Supplementary Material

Additional file 1 **Fraction of True Positives for different E-value bins for different co-complex networks**. Pdf-file containing a graph showing the fraction of True Positives for co-complex networks including links based on high-throughput data. Click here for file

Additional file 2 **Fraction of subunits found with BLAST or co-complex alignment, per complex**. An excel file containing for each complex the number and fraction of subunits found with BLAST or co-complex network alignment and how many of these subunits have been confirmed by either Pfam or PSI-BLAST. Click here for file

Additional file 3 **Per complex, for each subunit if we find a yeast homolog for this subunits, how we found it and if we could confirm the homology, how we confirmed it**. An excel file containing for each subunit from the human complexes we investigated, if we found a homolog in yeast for this subunit, how we found this homolog and how we confirmed it, and which yeast complex(es) the yeast homolog belongs to. Click here for file

Additional file 4 **Fraction of True Positives, normalized for family size for different E-value bins for different subsets of BLAST hits with that E-value**. Pdf-file containing a graph showing the fraction of True Positives when normalized per query protein. Click here for file
