# Supplementary material for: Enrichment of homologs in insignificant BLAST hits by co-complex network alignment
Source: BMC Bioinformatics. 2010 Feb 12;11:86. doi: 10.1186/1471-2105-11-86 (PMC2836305; doi:10.1186/1471-2105-11-86)
Supplement: Additional file 1 — Fraction of True Positives for different E-value bins for different co-complex networks. Pdf-file containing a graph showing the fraction of True Positives for co-complex networks including links based on high-throughput data. [file 1471-2105-11-86-S1.PDF]

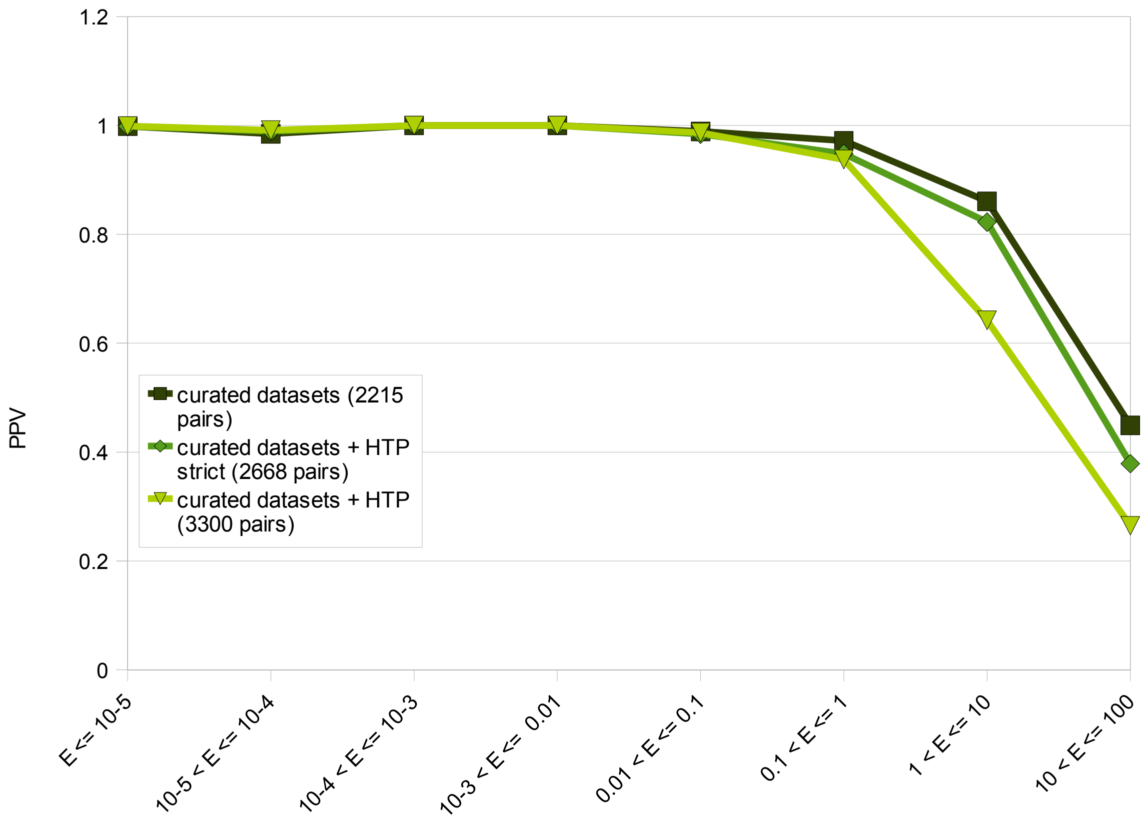

Additional file 1 - Fraction of True Positives for different E-value bins for different co-complex networks.

If we use only curated complex datasets to construct the co-complex networks we subsequently align, we get the largest overepresentation of True Positives (BLAST hits that are homologous according to Pfam), but low coverage (dark green line). We increase the coverage by including high-throughput co-complex data from large scale Tandem Affinity Purification Experiments, using a strict cutoff (yeast: confidence score > 0.3, human: confidence score > 0.5, green line) and using an inclusive strict cutoff (yeast: confidence score > 0.1, human: confidence score > 0.25, light green line) at a small cost in terms of performance.
