# Supplementary material for: Enrichment of homologs in insignificant BLAST hits by co-complex network alignment
Source: BMC Bioinformatics. 2010 Feb 12;11:86. doi: 10.1186/1471-2105-11-86 (PMC2836305; doi:10.1186/1471-2105-11-86)
Supplement: Additional file 4 — Fraction of True Positives, normalized for family size for different E-value bins for different subsets of BLAST hits with that E-value. Pdf-file containing a graph showing the fraction of True Positives when normalized per query protein. [file 1471-2105-11-86-S4.PDF]

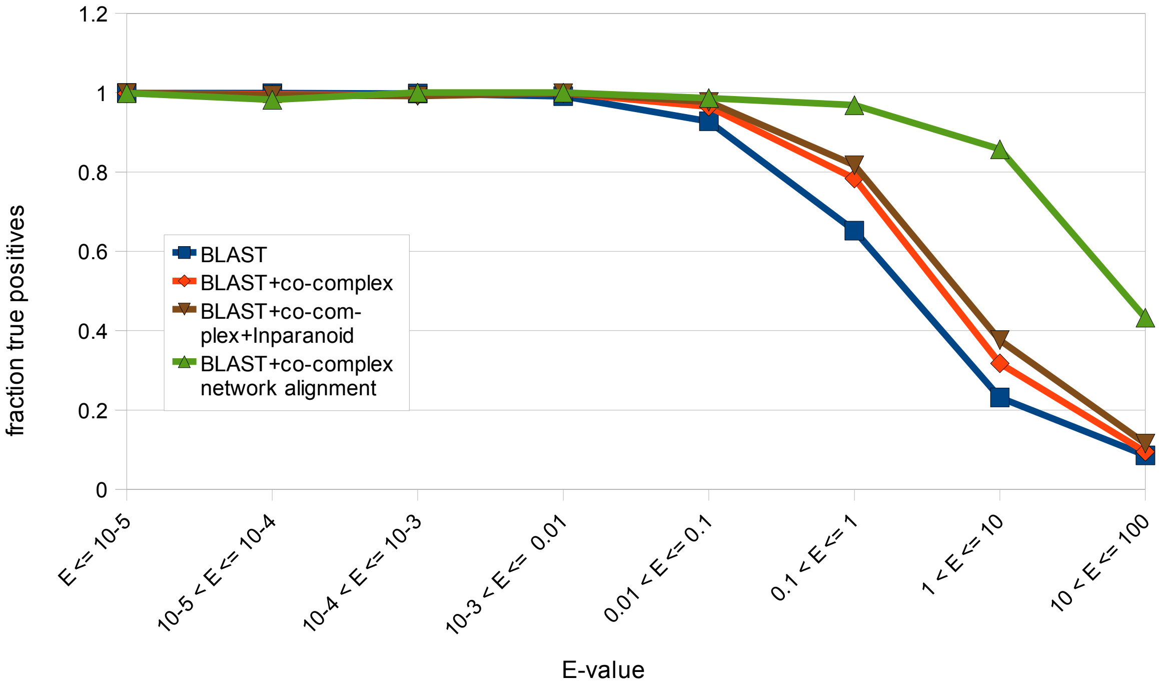

Additional file 4 - Fraction of True Positives, normalized for family size for different E-value bins for different subsets of BLAST hits with that E-value.

The fraction of True Positives, normalized per query, for all BLAST hits ('BLAST', blue line), the BLAST hits for which both the human query as the yeast hit are part of a co-complex network ('BLAST+cocomplex', red line), the BLAST hits for which both the human query as the yeast hit are part of a co-complex network and both have a direct co-complex network neighbor that has a clear ortholog in the other species (is part of a human-yeast Inparanoid cluster) ('BLAST+cocomplex+inparanoid', brown line), the BLAST hits for which both the human query as the yeast hit are part of a co-complex network and both have a direct co-complex network neighbor and these neighbors are clear orthologs of each other (are part of *the same* human-yeast Inparanoid cluster) ('BLAST+network alignment', green line). We normalized by taking the average fraction of True Positives per query.
